# Development and evaluation of an anteriorly mounted microprocessor-controlled powered hip joint prosthesis

**DOI:** 10.33137/cpoj.v7i2.44494

**Published:** 2025-02-07

**Authors:** K Brannen, N Baddour, L Cho, D Langlois, P Dumond, E.D Lemaire

**Affiliations:** 1 Department of Mechanical Engineering, Faculty of Engineering, University of Ottawa, Ottawa, Canada.; 2 Össur, Grjothals 1–5, 110 Reykjavik, Iceland.; 3 Department of Medicine, Faculty of Medicine, University of Ottawa, Ottawa, Canada.; 4 Center for Rehabilitation Research and Development, Ottawa Hospital Research Institute, Ottawa, Canada.

**Keywords:** Microprocessor-Controlled Prosthesis, Powered Hip Joint, Hip Disarticulation, Hemipelvectomy, Prosthesis Design, Amputation, Lower Limb Amputation, Hip–Knee–Ankle–Foot Prosthesis

## Abstract

**BACKGROUND:**

Prosthetic solutions for individuals with hip disarticulation and hemipelvectomy amputations currently rely exclusively on passive hip joint mechanisms. Although powered knee and ankle joint prostheses have improved gait in people with amputation, no powered hip joint options are commercially available.

**OBJECTIVE:**

To develop and validate the mechanism, structural integrity, and design of an anteriorly mounted powered hip joint prosthesis.

**METHODOLOGY:**

A microprocessor-controlled powered hip joint prosthesis (PHP) was developed, incorporating a cable-and-pulley transmission system. Stress calculations and Finite Element Analysis (FEA) were performed to ensure that the device can withstand the forces from daily activities. The prototype underwent mechanical strength testing in accordance with International Organization for Standardization (ISO) 15032:2000 standards, ensuring suitability for user loads of up to 100 kg. For functional testing, three able-bodied individuals were video recorded while walking with the power hip in a prosthesis simulator. For each participant, hip angles and stride parameters during level walking were assessed by analyzing five gait cycles.

**FINDINGS:**

The novel PHP met most of the design criteria; however, it protruded 56 mm anteriorly from the lamination plate, exceeding the specified criterion of 20 mm. The joint's range of motion included 22° of extension and 145° of flexion. The joint prototype's height was 347 mm, and it weighed 3.9 kg. Furthermore, it passed ISO 15032:2000 strength tests, withstanding a 3360 Newton (N) load without failure. The device successfully enabled able-bodied individuals to walk using a hip disarticulation simulator and supported a 98 kg user during level walking.

**CONCLUSION:**

The microprocessor-controlled PHP exhibited successful performance in both mechanical strength and functional testing. Future work is needed to optimize and assess the design, which could reduce the device's weight and size. A complex control system to adjust gait based on pelvic motion is currently under development.

## INTRODUCTION

Lower limb prostheses facilitate daily ambulation for people with amputation. Microprocessor powered lower limb prosthetics technology, primarily for knee joints, can improve balance and stability^1^ and can lower energy expenditure, leading to increased daily activity and overall satisfaction.^2^ However, similar solutions for hip joints are not available. Considering that walking performance is greatly affected by proximal amputation locations,^3^ many people with hip disarticulation (HD) and hemipelvectomy (HP) amputation experience difficulty walking with prostheses. As a result, more than 50% of such individuals opt for alternative solutions such as wheelchairs for daily mobility.^4^

A powered prosthetic hip joint could solve common challenges in current hip-knee-ankle-foot (HKAF) prostheses, namely stability, metabolic cost, and range of motion, allowing people to recover a more natural gait.^5^ Commonly used prosthetic hip joints can be grouped into single-axis or polycentric joints, based on the number of pivot points and linkages involved in the mechanism. While polycentric joints like the Ottobock Helix 3D have improved gait compared to other devices,^4^ any passive hip joint requires the user to generate hip moments by using lumbar spine and pelvis movements for propulsion. This comes with the disadvantage of high energy expenditure while walking,^3–6^ asymmetrical gait,^7^ and an increased risk of long-term injury from musculoskeletal imbalances.^8^

Microprocessor-controlled knee joints have better capability to react to user movement, detecting harmful movements like trips and stumbles to reduce injury risk.^9^ These devices can also adapt to various walking environments (e.g., level ground, ramp, stairs). Given that the average adult performs approximately 60 sit-to-stand movements daily,^10^ the added support and assistance from a powered prosthesis could reduce sit-stand asymmetry and load on the intact leg in individuals with amputation. A powered prosthetic hip joint could enable step-over-step stair ascent, a capability currently lacking in people with HD and HP amputation.^11^

A powered prosthetic hip joint was reported by Ueyama et al.,^12^ who prototyped a device using direct current (DC) motors positioned within the thigh to power the hip and knee joints, with the joints sharing microcontroller boards, battery, and sensor data. An able-bodied participant successfully walked with the prosthesis, but the device required users to wear the battery in a waist bag and the prosthesis would not fit under clothing.

A laterally mounted powered hip joint prosthesis was previously developed by Mroz et al.^13^ with the hip axis of rotation similar to the anatomical joint center. A cable driven transmission system transferred motor torque from the prosthetic thigh to the hip axis of rotation. The thigh chassis that housed the battery and electronics was separately validated.^14^ A weight bearing strut with double row steel ball bearings provided clearance for the device to swing beneath the pelvis.^13^ Able-bodied participants successfully walked with the laterally mounted PHP using a prosthesis simulator. Although this prototype successfully met most of the design requirements, there is still room for improvement in terms of weight and size.

This paper explored the design and development of the main mechanical and structural components of an anteriorly mounted PHP, focusing on the rope and pulley transmission system that drives the mechanism. The anteriorly mounted design provides a compact system with the motor and electronics in the thigh, distal to the joint center of rotation. A successful PHP could greatly enhance safe mobility for people with HD or HP amputations.

## METHODOLOGY

### Design Requirements

Based on existing international standards, literature review, the research team's clinical experience working with HD, HP or transfemoral amputation, and discussions with experts, the following design criteria were selected:

- Joint angular velocity of at least 150 °/s.^15,16^
- Maximum hip moment of 96 Nm to accommodate for a 100 kg user.^17,18^
- Joint range of motion of 130° flexion and 20° extension.^18^
- Device weight should remain under 4 kg. This is compared to Össur's Power Knee weighing 3.2 kg using the same actuator and battery.^19^
- Device strength based on ISO 15032:2000 standards for prosthetic hip joints:^20^
  - Withstand 2240 N load for 30 s without deformation >15 mm, and 3360 N without ductile failure.
  - Joint must withstand 2x106 cycles from 50 N to 1330 N without failure.
- Must comfortably fit under user clothing:
  - Anterior protrusion less than 20 mm from the center of lamination plate.
  - Lateral protrusion less than 80 mm from center of lamination plate.^21^
  - Medial protrusion less than 50 mm from center of lamination plate.
- Device length must remain under 378 mm (using anthropometric data, thigh length is 0.245*height, or 378 mm for the 15th percentile women height).^21^
- No finger traps or sharp edges for user safety.

### Design and development of PHP prototype

**Figure 1:**
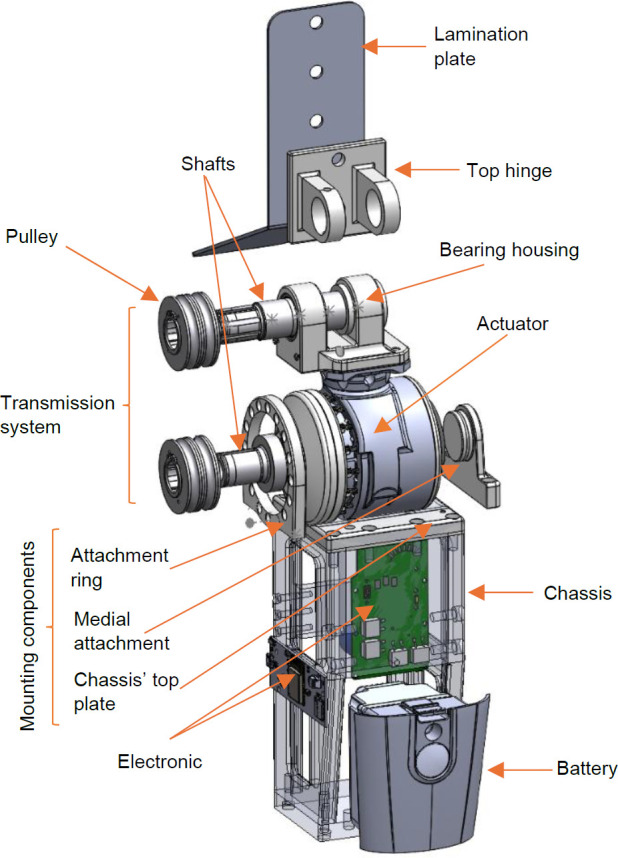
Powered hip joint components. Transmission system: Drive train composed of four pulleys of equal diameter and steel cables enabling joint flexion and extension (steel cables are not shown in this figure); Shafts: Structural components that bear user weight and tension from the pulleys. The proximal shaft connects the top hinge and bearing housing, and the distal shaft applies torque to the bottom pulleys; Bearing housing: Connects to the top of the actuator and facilitates joint rotation around the top shaft; Top hinge: Interface between the lamination plate and joint center; Lamination plate: Plate embedded in the prosthetic socket to attach the joint; Actuator: Össur Power Knee™ microprocessor-controlled motor modified for the PHP, providing torque and power to the system; Mounting components: Attachment ring and medial attachment piece secures the actuator to the attachment plate (chassis' top plate); Chassis: Contains the electronic and the battery (Össur Power Knee).

The PHP main components (**Figure 1**) were designed using SolidWorks software (Version 2020). All metal components were made with aluminum 2024-T4 or 17-4 PH stainless steel, except for the off-the-shelf parts like screws and retaining rings. To make the prototype lighter, aluminum was used as much as possible, but steel was needed for parts requiring more strength. Note that the actuator is located below the joint to minimize anterior protrusion. Actuator torque is transmitted through the cable and pulley to the joint center.^22^

**Figure 2:**
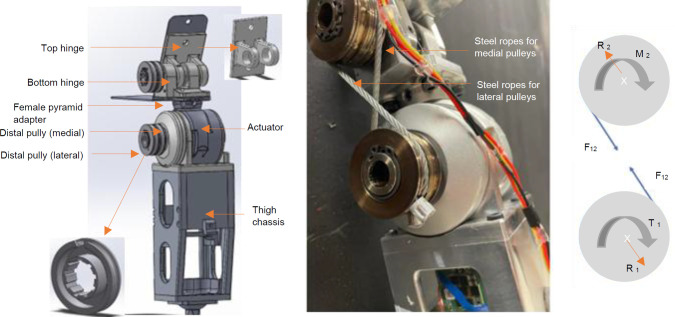
Left: Schematic of the PHP and a pulley; Middle: Illustration of the steel ropes and pulleys; Right: Free body diagram of the crossed cable system, where T_1_ is the input torque from the actuator acting on the driving pulley, F_12_ is the tensile force from the cables on the pulleys, M_2_ is the reaction moment on the fixed pulley shaft, R_1_, and R_2_ are the reaction force of the cable force on the pulleys.

The cable and pulley transmission system consists of four equally sized pulleys connected by two high strength steel cables (**Figure 2**). When torque is applied by the actuator to the bottom pulley, tension is applied to the cable and the assembly rotates around the top pulley in the opposite direction. Much like a belt drive transmission, the mechanism transfers torque over an extended distance with less components and weight. Pulleys diameters are also constrained since a small diameter requires greater tension on the cable to achieve the same torque (**Figure 2**). Therefore, 52 mm diameter pulleys were chosen to maintain the system's integrity while balancing performance and compactness.

The cables were crossed in a figure-eight configuration, to ensure that the input torque from the motor and the reaction torque from the fixed pulleys (top) are in the same direction. Without a counteracting torque to establish equilibrium, the entire system rotates. Pulleys of equal diameter were selected, ensuring equal speed and torque between the top and bottom pulleys, to deliver the required 96 Nm of torque and 150°/s angular velocity. The pulleys were machined from 17-4 PH H900 steel to withstand high radial loads imposed by the cable and were designed to have a minimal diameter to reduce weight and space. The PHP pulleys were designed to allow the rope to anchor inside the part, to prevent slipping (**Figure 2**). The rope loop fits around a groove on the inside of the pulley and a hole on the top allows the rope to stick out and wrap around the pulley.

High strength ropes or cables are required to meet the load requirements and provide better flexibility, and greater strength. To achieve a 96 Nm hip moment with a 52 mm diameter pulley, 4200 N of tension must be applied to the rope. Liquid crystal polymer (LCP) ropes such as Vectran™ (https://kuraray.us.com/products/fibers/vectran/) meet these requirements, but steel cabling was selected for the initial prototype testing due to ease of crimping on terminators (**Figure 2**).

The top hinge is secured to both the top shaft and lamination plate (**Figure 1**), while the bottom hinge is mounted on bearings. The bearing housing connects to a female pyramid adapter that is secured to its male counterpart on the actuator (**Figure 2**). Needle roller bearings were selected for this application for their ability to handle high radial loads while occupying minimal space.

**Figure 3:**
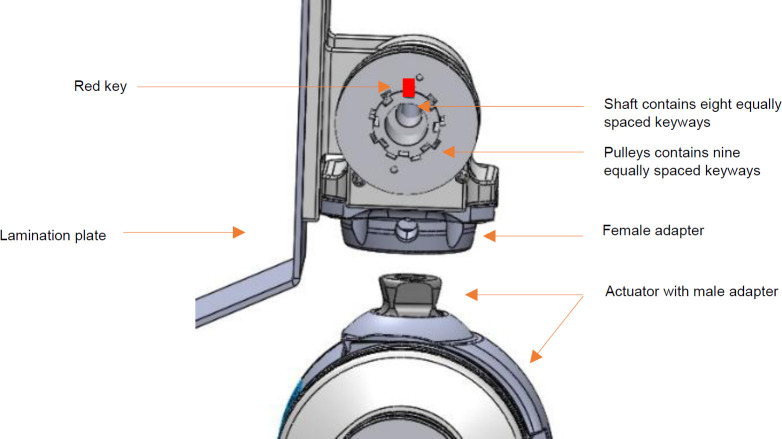
Offset keyway tensioning solution. The red key demonstrates one of the pulley positioning possibilities.

Like many transmission systems, the cable and pulley system must be pre-tensioned to ensure control responsiveness. If slack exists at an idle position, the actuator must first turn to take up the slack before joint movement can occur. For optimal performance, the joint must rotate synchronously with the actuator to avoid backlash caused by the oscillating nature of the mechanism. This issue was addressed by implementing a novel tensioning system consisting of multiple keyways on the top shaft and pulleys. The shaft contains eight equally spaced keyways, while the pulleys contain nine. This results in 72 possible orientations of the top pulleys where the top pulleys can be rotated and secured every 5° via a compatible keyway (**Figure 3**). Additionally, the system requires the medial and lateral pulleys to be tensioned in opposite directions, necessitating the use of two separate pulleys on the same shaft.

**Figure 4:**
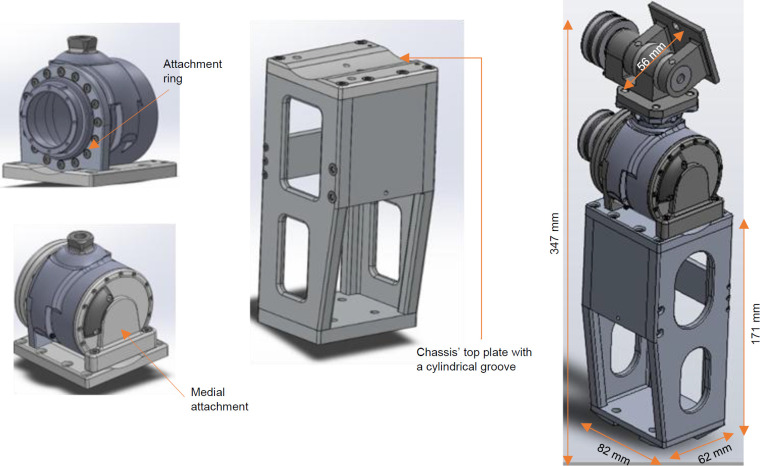
Left: Attachment ring and medial attachment piece bracing the outer casing of the actuator to the top plate of the electronics chassis; Middel: The thigh chassis that housed the battery and electronics; Right: Joint dimensions.

Two main mechanisms were used to mount the PHP to the top plate of an aluminum chassis that holds the battery and other electrical components (**Figure 4**). A cylindrical groove was machined out of the chassis' top plate to ensure full contact with the actuator's bottom. The attachment ring contains a circular bolt pattern that fastens the ring to the circumference of the actuator. Two tapped holes were located underneath the attachment ring to secure the ring to the chassis' top plate, restricting movement of the actuator's outer casing. An additional medial attachment piece was fastened to the medial side of the actuator's center, providing a secondary bracing mechanism to prevent the actuator from lifting off the chassis.

The PHP prototype was constructed from an assembly of machined parts and off the shelf components. Mechanical drawings with appropriate dimensions and tolerances for each part were made before machining.

An Össur Power Knee™ microprocessor-controlled motor was modified for the PHP, providing torque and power to the system. The lamination plate was designed to fit a prosthesis simulator that allowed able bodied participants to test the prototype.^23^

### Testing and Validation

#### Static Load Testing

**Figure 5:**
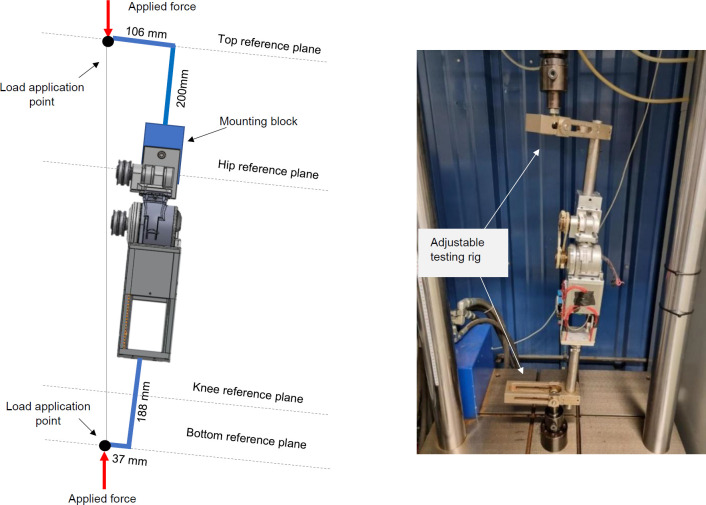
Left: Medial-Lateral mechanical testing (ISO-15032:2000) conditions for the powered hip prosthesis; Right: Setup for static load.

**Figure 6:**
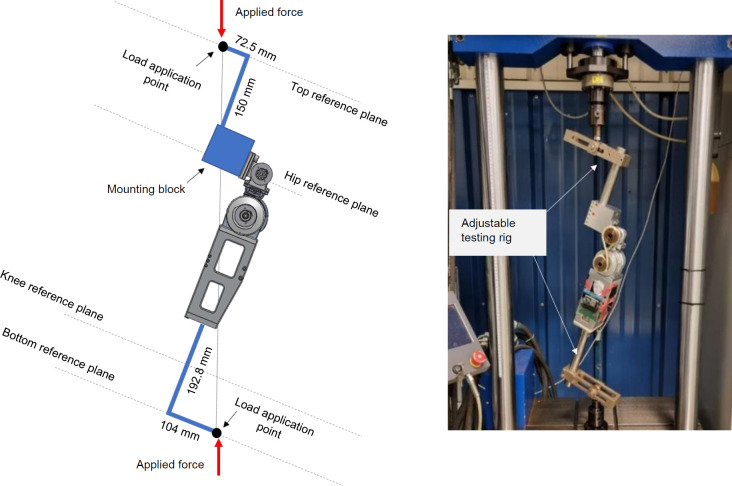
Left: Anterior-posterior mechanical testing (ISO-15032:2000) conditions for the powered hip prosthesis; Right: Setup for static load.

Static load testing ensured that the PHP was strong enough to withstand operating loads of a 100 kg person, using the procedures outlined in ISO 15032:2000 Prostheses – Structural testing of hip joints.^20^ Two testing conditions were evaluated: medial-lateral (ML) and anterior-posterior (AP) extension (**Figure 5** and **Figure 6**). The PHP was tested in a servo hydraulic testing system. This study only explores prototype functionality under short-term use.

Since the servo hydraulic testing system could only move along one axis, an adjustable testing rig was developed. The PHP top hinge was bolted onto an aluminum block attached to a pole and adjustable moment arm. The bottom of the chassis was attached to a similar assembly by a pyramid adapter. The testing procedure was as follows:

1. Set force to a 1024 N settling load and hold for 30 s.
2. Return load to zero.
3. Increase load at a rate of 200 N/s until 3360 N.
4. Return load to zero.

The ML test was set up in the fully extended position, as shown in **Figure 5**. All moment arm lengths and angles were based on the ISO 15032:200 0 medial lateral loading conditions.

#### Functional Testing

The functional testing protocol was approved by the University of Ottawa Office of Research Ethics and Integrity. Functional testing was performed by three able-bodied participants (members of the research team) on a prosthesis simulator^23^ that enables them to walk on a HKAF prosthesis. Informed consent was obtained from all participants, and the inclusion criterion was being able-bodied without any balance issues.

**Figure 7:**
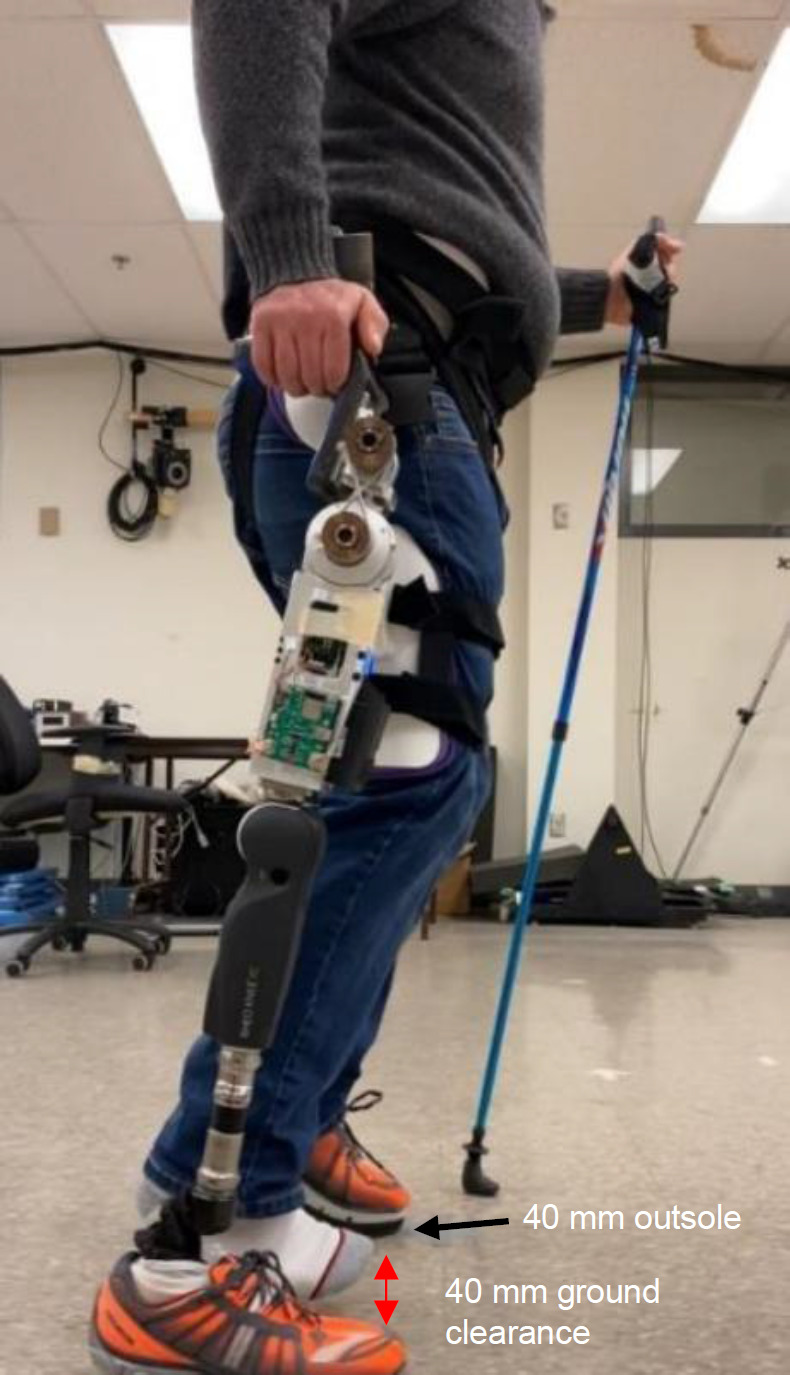
Hip disarticulation simulator^23^ setup with PHP, Össur Rheo 3 knee joint, and Össur Pro-Flex XC foot. Participants wore an elevated outsole on the intact limb to create 40 mm ground clearance for the right foot during stance.

The assembly consisted of the PHP, Össur Rheo 3 knee joint, and Össur Pro-Flex XC foot. Participants wore an elevated outsole on the intact limb (left leg) to create 40 mm ground clearance for the right foot during stance (**Figure 7**). In this setup, only the prosthesis simulator and the left leg contacted the ground.^23^ Participant subjective feedback was also recorded comparing the PHP to previous Helix3D walking tests. Each participant was first trained on the simulator using the OttoBock Helix 3D joint and learned to walk comfortably before continuing with trials using the PHP. One or two canes were also used by the participants for support and safety. The participants were given one training session to familiarize themselves to walking with the PHP on the simulator and one testing session where the data was recorded.

For functional testing, a simple control system was implemented for the PHP. It applied a predetermined and repeated gait cycle, tuned to operate at a specific pace to provide a comfortable gait pattern for all users. This cycle began at 40° flexion at heel strike and then extended to 20° extension. Once full extension was reached, the joint swung to 44° flexion and slowly returned to the initial condition of 40° flexion. A 2.5 s stride time was implemented to suit the participants. PHP gait profile used for functional testing was illustrated in the authors' previous publication.^13^

For functional testing, five gait cycles were video recorded using a smartphone where no large stumbles were present and where the participant did not contact the floor with their prosthetic side natural foot. The videos were analyzed using the Kinovea video annotation tool to measure the hip angle (angle between the torso and the line connecting the hip joint and the knee) and determine stride parameters. Initial contact and toe-off times were used to identify the step times of each stride for each participant.

## RESULTS

Successful construction of the prototype validated the PHP manufacturing and assembly methods. The device weighed a total 3.9 kg, including the battery, electronics, and chassis. This prototype, similar to the Ottobock Helix 3D, features right and left configurations. In this study, a right PHP was manufactured and tested.

### Static load test results

**Figure 8:**
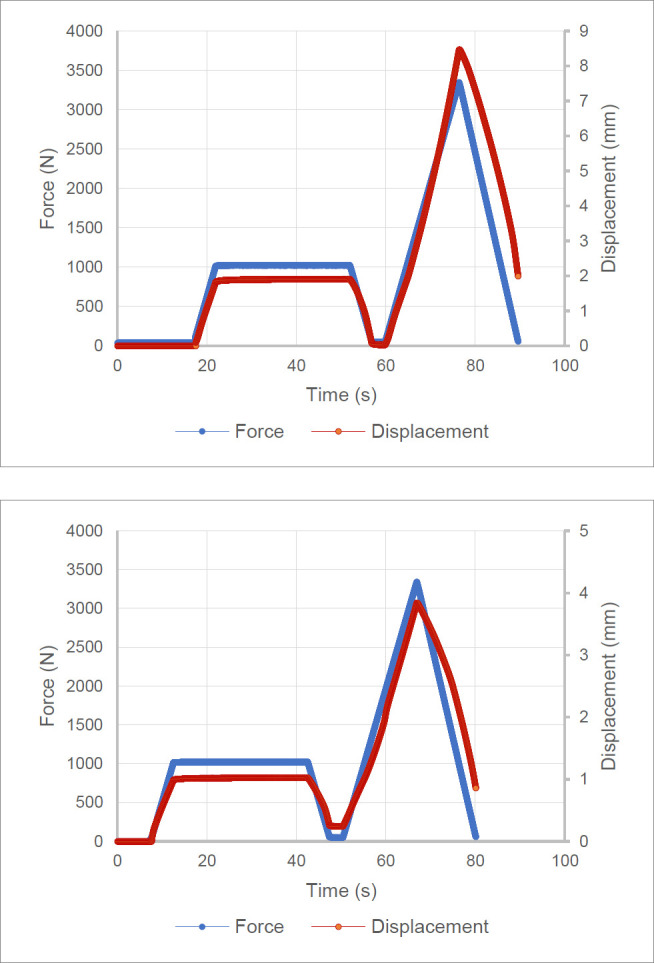
Top: PHP force and displacement profiles for medial-lateral static load testing; Bottom: PHP force and displacement profiles for anterior-posterior static load testing.

The test procedure was the same for both ML and AP, with the only difference being the loading conditions. **Figure 8** depict the force and displacement versus time profiles of the ML test and AP tests, respectively. Both tests withstood the 3360 N ultimate strength test, displaying no signs of ductile fracture or plastic deformation. The ML test displayed a maximum displacement of 8.5 mm, whereas the AP test displayed less displacement at 4 mm (**Figure 8**).

### Functional testing results

**Figure 9:**
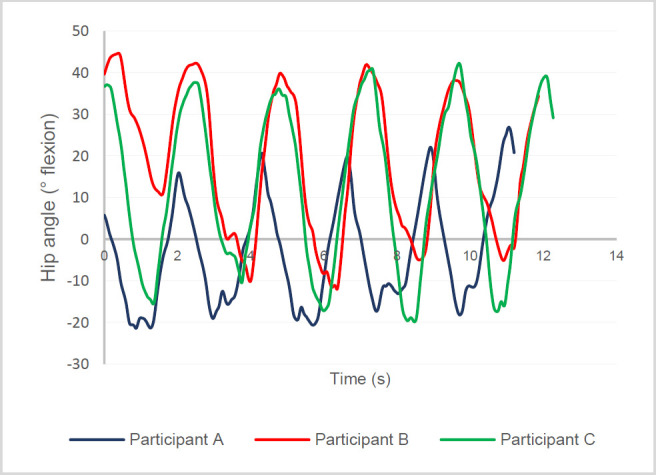
PHP hip flexion angle vs time across 5 strides for participant A (blue), participant B (red), and participant C (green).

Three male volunteers (members of the research team) participated in functional testing (**Table 1**). Heel contact and toe-off times were used to identify the step times of each stride for each participant. The results of the five-stride test are shown in **Figure 9**.

**Table 1:** Participant information.

Participant	A	B	C
**Sex**	Male	Male	Male
**Age (years)**	44	28	25
**Height (cm)**	178	180	175
**Weight (kg)**	95	95	98
**Number of canes used by participants**	1	2	2

Maximum flexion and extension angles were identified in **Table 2**. The average range of motion during level walking was 47.2 ± 6.4°, which remained within the sufficient preprogrammed range of 64°.

**Table 2:** Functional testing range of motion.

Participant	Maximum flexion (°)	Maximum extension (°)	Range of motion (°)
A	21.1 ± 4.0	19.3 ± 1.7	40.4 ± 4.3
B	41.4 ± 2.5	4.3 ± 8.9	45.7 ± 9.2
C	39.3 ± 2.5	16.1 ± 3.5	55.3 ± 4.3
Average	33.9 ± 3.1	13.2 ± 5.6	47.2 ± 6.4

The average step time on the prosthetic side averaged 0.63 times the intact side. The average swing time for the intact side was 0.29 ± 0.04 s and the average prosthetic leg swing time was 1.08 ± 0.10 s (**Table 3**).

**Table 3:** Functional testing gait parameters with percentages of average stride time.

Participant		A	B	C	Average
**Stride time (s)**		2.35 ± 0.07	2.38 ± 0.10	2.45 ± 0.09	2.39 ± 0.09
**Step time (s)**	**Prosthetic**	1.50 ± 0.10 (64 %)	1.19 ± 0.06 (50%)	1.24 ± 0.08 (50%)	1.31 ± 0.08 (55%)
	**Intact**	2.04 ± 0.06 (88%)	2.09 ± 0.02 (89%)	2.09 ± 0.10 (86%)	2.07 ± 0.07 (88%)
**Swing time (s)**	**Prosthetic**	0.85 ± 0.07 (36%)	1.18 ± 0.14 (50%)	1.22 ± 0.08 (50%)	1.08 ± 0.10 (45%)
	**Intact**	0.27 ± 0.02 (12%)	0.27 ± 0.03 (11%)	0.34 ± 0.05 (14%)	0.29 ± 0.04 (12%)
**Double support time (s)**		0.94 ± 0.16 (40%)	0.91 ± 0.08 (38%)	0.89 ± 0.06 (36%)	0.91 ± 0.11 (38%)
**Cadence (steps/min)**		51.2 ± 1.4	50.6 ± 2.2	49.0 ± 1.9	50.3 ± 1.8
**Step time ratio**		0.73 ± 0.05	0.57 ± 0.03	0.59 ± 0.06	0.63 ± 0.05

All three participants stated that the PHP was easier to operate than the non-powered Helix3D joint, specifically noting that manual swinging and large pelvic rotation were not necessary for propulsion (subjective feedback). The participants also noted that the additional weight of the PHP compared to the Helix3D did not cause any problems.

### Design Requirements Evaluation

The final design tested in this study met most of the design criteria (**Table 4**).

**Table 4:** Design criteria and results.

	Requirement	Actual value	Met the design criteria
**Device weight**	Maximum 4.0 kg	Measured 3.9 kg	Yes
**User weight**	Maximum 100 kg	Tested with 98 kg user Passed strength tests for 100 kg user	Yes
**Strength**	Withstand 2240 N load for 30 s without failure or deformation > 15 mm Withstand 3360 N load without ductile failure Withstand 2×10^6^ cycles between 50 N and 1330 N without failure	Passed static loading tests FEA simulations indicated that device should pass cyclical loading test	Yes
**Range of motion**	Minimum 20° hip extension	22° hip extension (measured with protractor)	Yes
	Minimum 130° hip flexion	145° hip flexion (measured with protractor)	Yes
**Hip moment**	Minimum 96 Nm	1:1 gear ratio should provide 96 Nm hip moment	Yes*
**Angular velocity**	Minimum 150 °/s	1:1 gear ratio should provide 300°/s angular velocity	Yes*
**Anterior protrusion**	Maximum 20 mm from top of lamination plate	56 mm (measured with ruler) without the cover-59 mm with the cover	No
**Lateral protrusion**	Maximum 7.99 cm from centre of lamination plate	7.2 cm (measured with ruler)	Yes
**Medial protrusion**	Maximum 4.99 cm from centre of lamination plate	4.7 cm (measured with ruler)	Yes
**Device length**	Maximum 378 mm	347 mm (measured with ruler)	Yes
**User safety**	No uncovered finger traps	Cover prevents most finger traps Cover does not fit over steel cables	No

* The Össur Power Knee™ microprocessor-controlled motor was used in this prototype, which provides 96 Nm of hip torque and an angular velocity of 300°/s.

## DISCUSSION

A novel microprocessor-controlled PHP was designed and evaluated for both strength and function. The pulley and cable power transmission system was successful in transmitting rotational power from the actuator to the hip joint. The final design met most of the design criteria, including mechanical strength tests. The PHP was also tested with able-bodied participants using a hip disarticulation prosthesis simulator, where ambulation was successful.

The device weighed 3.9 kg, putting the PHP under the 4.0 kg weight limit. The functional testing participants noted that this 3.9 kg weight did not feel heavy while walking or noticeably impede their motion. The anterior protrusion criterion was 20 mm, but the prototype tested in this study protruded 56 mm. Future work is needed to optimize and assess the design, which could reduce the device's weight and size.

The design requirement for the PHP was to support users up to 100 kg. The PHP successfully supported a 98 kg user during functional testing, which is close to the required 100 kg. The PHP also successfully passed ISO mechanical testing designed for users up to 100 kg. The strength requirements outlined in ISO 15032:2000 were to withstand a 2240 N load for 30 s without failure or deformation greater than 15 mm, withstand a 3360 N load without ductile failure, and withstand 2×106 cycles between 50 N and 1330 N without failure. The PHP withstood a 3360 N load without failure or deformation greater than 15 mm. Cyclical testing was not conducted on the PHP; however, calculations and FEA simulations indicate that the device should be able to withstand fatigue from the cycles outlined in the ISO standard.

During level walking, the PHP achieved an average of 13.2 ± 5.6° of extension and 33.9 ± 3.1° of flexion (**Figure 9**). The maximum extension and flexion measured with a protractor were 22° and 145°, respectively, successfully surpassing the 20° hip extension and 130° hip flexion requirements.

The final PHP power transmission gear ratio was 1:1. Therefore, the device should have the same maximum torque and angular velocity as the Össur Power Knee™ microprocessor-controlled motor. The maximum actuator torque is 96 Nm and the maximum angular velocity is 300°/s, reaching the outlined 96 Nm and 150°/s outlined criteria. The actual hip moment was not measured; however, moments were enough to successfully propel all three functional testing participants forward and support body weight, allowing level ground walking.

Geometric constraints were established to ensure the PHP could fit comfortably under a user's pants. The first geometric restriction was that the PHP could not protrude more than 20 mm from the top of the lamination plate. The final prototype for initial testing protruded 56 mm anteriorly, failing to meet the criterion. Even though this criterion not met, this prototype could still be accommodated under loose-fitting pants. The PHP could also not protrude more than 80 mm laterally or 50 mm medially from the center of the lamination plate. 71 mm lateral protrusion and 50 mm medial protrusion were measured on the final device. The device length was also controlled to ensure a large population could use the device.

The PHP could not have any uncovered finger traps. A cover was designed to go over the pulley system, where the main finger traps occurred. However, the cover no longer fit over the pulleys when the steel cables were used for functional testing. A cover that encompasses the entire device would also be more successful because there is still potential for a finger to be caught between the bearing housing and the lamination plate with the current design. Therefore, this requirement was only partially met and could be improved upon.

From the functional test results, discrepancies in the joint range of motion data were present, where the recorded range of motion was 8.3° less than the joint's pre-programed range of motion. This difference was likely the result of a difference in step timing (landing early) compared to the pre-programmed gait profile. Offsets in the recording angle can cause inaccuracies in the measurements since the footage was recorded in 2D. For future work, biomechanical data should be collected on people with HD or HP amputations, using 3D motion capture systems to accurately measure gait data.

Another key finding from the functional test results is that the user tends to spend more time on their intact leg compared to the prosthesis. This causes asymmetrical gait patterns and is likely the result of the user feeling less stable on the prosthetic side. To be more specific, when the participant is supported with the prosthesis, they will quickly swing their intact leg in front for support. Feelings of discomfort and instability may be reduced with more training and experience with the prosthesis, along with a fully developed intelligent control system.

The PHP tested in the current study weighs 3.9 kg, meeting the design criteria. Ueyama et al.,^12^ who prototyped a device using direct current (DC) motors, also noted that developing a lightweight powered hip joint prosthesis is challenging. They reported that the socket weighed 1.5 kg, resulting in a total robotic HDP weight of 11.3 kg. They did not provide the joint's weight separately. As well, electronics and battery were located at the waist, not integrated into the prosthesis. Similarly, Mroz et al.,^13^ who evaluated a PHP mounted laterally, mentioned weight as a challenge, with their prototype weighing 5.7 kg. Further work is needed to decrease the PHP's size and weight.

### Limitations

There are some limitations in this study. Since the control system was not fully developed during the primary functional testing, a simple control system was implemented that could only be used for level walking at a fixed speed. Additionally, the study was conducted with only three able-bodied individuals. Evaluation with HD or HP is required in future evaluation.

During the initial testing, steel cabling was selected due to ease of crimping on terminators. However, steel cables are not ideal for a final product because their rated capacity is 1779 N, which falls below the design criterion tensile strength of 4465 N, posing a risk of failure under increased loads.

## CONCLUSION

The novel microprocessor-controlled PHP demonstrated successful performance in both mechanical strength and functional testing. The pulley and cable transmission system effectively transmitted power from the actuator to the hip joint, meeting the functional design requirements while maintaining a compact and lightweight profile, showing strong potential for real-world applications. While the PHP achieved most of its principal design objectives, some areas like part optimization, control system design, and cable strength and slack management require further refinement. Future iterations could focus on implementing an intelligent adaptive control system, optimizing mechanical parts and mechanisms, and continuing functional testing with people with HD or HP amputations.

At this stage of development, a reasonable balance between comfort and safety has been achieved and will be further refined as development progresses. Continued research and development will aim to address the remaining challenges, prioritizing gait stability and symmetry, pushing the technology closer to real-world deployment.

## DECLARATION OF CONFLICTING INTERESTS

David Langlois is an employee of Össur. No other conflicts of interest.

## AUTHORS CONTRIBUTION

- **Kelly Brannen:** Conceptualization; Joint design; Data collection and analysis; Manuscript revision.
- **Natalie Baddour:** Conceptualization; Data analysis; Manuscript revision, Supervision.
- **Lucas Cho:** Prepared the initial manuscript; Manuscript revision.
- **David Langlois:** Conceptualization; Design.
- **Patrick Dumond:** Design.
- **Edward Lemaire:** Conceptualization; Data analysis; Manuscript revision; Supervision.

## SOURCES OF SUPPORT

This study was financially supported by Össur and Mitacs.
